# Effect of Hydroxyapatite Formation on Titanium Surface with Bone Morphogenetic Protein-2 Loading through Electrochemical Deposition on MG-63 Cells

**DOI:** 10.3390/ma11101897

**Published:** 2018-10-04

**Authors:** Huei Yu Huang, Yankuba B. Manga, Wan-Ning Huang, Chung-Kwei Lin, Ching-Li Tseng, Haw-Ming Huang, Chia-Yu Wu, Chi-Chang Wu

**Affiliations:** 1 Department of Dentistry, Taipei Medical University-Shuang Ho Hospital, 23561 Taipei, Taiwan; hyhuang77@gmail.com; 2 School of Dentistry, College of Oral Medicine, Taipei Medical University, 11031 Taipei, Taiwan; borgiawu@gmail.com; 3 Graduate Institute of Biomedical Materials and Tissue Engineering, College of Biomedical Engineering, Taipei Medical University, 11031 Taipei, Taiwan; yanks201082@gmail.com (Y.B.M.); bow0917@gmail.com (W.-N.H.); chingli@tmu.edu.tw (C.-L.T.); 4 School of Dental Technology, College of Oral Medicine, Taipei Medical University, 11031 Taipei, Taiwan; chungkwei@tmu.edu.tw; 5 Graduate Institute of Biomedical Optomechatronics Engineering, College of Biomedical Engineering, Taipei Medical University, 11031 Taipei, Taiwan; hhm@tmu.edu.tw; 6 Department of Dentistry, Taipei Medical University Hospital, 11031 Taipei, Taiwan; 7 Department of Electronic Engineering, Feng Chia University, 40742 Taichung, Taiwan

**Keywords:** calcium phosphate, bone regeneration, electrochemical deposition, hydroxyapatite

## Abstract

Calcium phosphate ceramics used in dentistry and orthopedics are some of the most valuable biomaterials, owing to their excellent osteoconduction, osteoinduction, and osseointegration. Osteoconduction and osteoinduction are critical targets for bone regeneration, and osseointegration is essential for any dental implantations. In this study, a hydroxyapatite (HAp) hybrid coating layer with the sequential release of bone morphogenetic protein 2 (BMP-2) was deposited onto an etched titanium substrate by electrochemical deposition. The resulting release of BMP-2 from Ti–HAp was assessed by immersing samples in a simulated buffer fluid solution. Through coculture, human osteosarcoma cell proliferation and alkaline phosphatase activity were assessed. The characteristics and effect on cell proliferation of the hybrid coatings were investigated for their functionality through X-ray diffraction (XRD) and cell proliferation assays. Findings revealed that −0.8 V vs. Ag/AgCl (3 M KCl) exhibited the optimal HAp properties and a successfully coated HAp layer. XRD confirmed the crystallinity of the deposited HAp on the titanium surface. Ti-0.8 V Ti–HAp co-coating BMP sample exhibited the highest cell proliferation efficiency and was more favorable for cell growth. A successful biocompatible hybrid coating with optimized redox voltage enhanced the osseointegration process. The findings suggest that this technique could have promising clinical applications to enhance the healing times and success rates of dental implantation.

## 1. Introduction

The use of biomaterials in dental implants has been prevalent for many years. Bone integration is a key factor determining a successful denture installation. If a denture is installed before bone regeneration (i.e., osseointegration), the tendency for dental failure is higher. Biomaterials such as Titanium-Aluminum-Vanadium alloy (Ti–6Al–4V, UNS R56400) are widely used in dental implants because of their excellent mechanical properties, high corrosion resistance, and superior osteocompatibility [1,2,3]. Osseointegration can require 3–4 months. Thus, it is desirable to shorten the healing time by enhancing osseointegration between Ti–6Al–4V and alveolar bone. The acceleration of osseointegration would reduce the inconvenience and discomfort experienced by patients following dental implant procedures.

There are numerous techniques used for coating Ti substrates, such as electrochemical deposition (ECD) [4], sol–gel [5], and plasma spraying [6]. Plasma spray is the most commonly used Ti–6Al–4V surface coating technique for implant attachment and bone integration [7]. However, this coating method has significant drawbacks, such as coating porosity and residual stress at the coating interface, as well as drastic changes in the composition and crystallinity of the calcium phosphate (CaP) powder [8,9].

ECD is a rapid and straightforward method, and provides good control of the coating material’s thickness, uniformity, crystallinity, and stoichiometry [10]. The process temperature of ECD is low compared to the plasma spray method. This method is typically used to coat hydroxyapatite (HAp, Ca_5_(PO_4_)_3_OH), which is a widely used biomaterial for bone implants [11]. HAp is a naturally occurring mineral form belonging to the calcium phosphate (CaP) family [12]. Therefore, HAp modification using ECD method on Ti–6Al–4V surface could have favorable results for osteoconduction.

Recently, coating with a bioactive growth factor has been determined to enhance the relationship between an implant and the bone to which it is attached [13]. Growth factors play crucial roles in various processes. Typically, they act as signaling molecules regulating bone induction, cell proliferation, as well as chondrocyte and osteoblast synthesis [14]. Some of these factors may affect osseointegration by altering the implant material, shape, surface texture, and hydrophilicity [15]. Thus, due to hydrophilicity, all dental implant surfaces must be treated to promote cell attachment [16]. However, if produced at a high temperature, bioactive coatings on the surface of Ti–6Al–4V can result in a weaker CaP phase and consequent peeling from the deeper coating layers [8]. In implants, bioactivity, bone resorption drugs, and growth factors may efficiently improve bone integration [17]. Among the growth factors (including platelet-derived growth factor, vascular endothelial growth factor, and transforming growth factor beta), BMP-2 is the most stable and least complicated [18]. BMP-2 enhances the osteogenic activity of osteocytes, accelerates osteoblast differentiation, and further promotes bone formation. It can also induce dentin and post-implant healing [19]. Free BMP-2 in blood is reported to rapidly spread to unrepaired blood vessels and surrounding tissue at the injury site [20]. BMP-2 can also increase osteogenic activity and stimulates bone-induced cell media, and bone defects can be repaired through stimulation by mesenchymal stem cells differentiating into osteoblasts, which induce dentin [21,22].

Jenny et al. reported that in the ECD process, the crystallization of calcium phosphate dehydrate and drug release rate were strongly dependent on the amount of carboxymethyl hexanoyl chitosan [23]. However, that paper lacks a discussion of the influence of applied ECD voltage on the material characteristics of the coated HAp film, as well as its osseointegration properties in cell test. Therefore, in this paper, the properties of HAp films using ECD coating at various application voltages were investigated. BMP-2 co-precipitated with HAp on the Ti–6Al–4V at different voltages was also studied to check its osteoconductivity, osteoinductivity, and osseointegration.

## 2. Materials and Methods

The Ti–6Al–4V specimens (10 mm × 10 mm × 1 mm) were purchased from Gloria Material Technology Corp. (Taipei, Taiwan). Analytical-grade hydrochloric acid (HCl, 33%), sodium bicarbonate (NaHCO_3_, 84.01 g/mol), potassium chloride (KCl, 74.56 g/mol), potassium phosphate dibasic trihydrate (K_2_HPO_4_·3H_2_O, 228.22 g/mol), magnesium chloride hexahydrate (MgCl_2_·6H_2_O, 203.31 g/mol), sodium sulfate (Na_2_SO_4_, 142.04 g/mol), calcium nitrate tetrahydrate (Ca(NO_3_)_2_·4H_2_O, 236.15 g/mol), recombinant human BMP-2, MTT (3-(4,5-dimethylthiazol-2-yl)-2,5-diphenyl tetrazolium bromide, C_18_H_17_N_5_S, 335.43 g/mol), and dimethyl sulfoxide (DMSO, (CH_3_)_2_SO, 78.13 g/mol) were purchased from Sigma-Aldrich (St. Louis, MO, USA). Analytical-grade sulfuric acid (H_2_SO_4_, 98%) was purchased from AENCORE (Victoria, Australia). Sodium chloride crystal (NaCl, 58.44 g/mol), calcium chloride pellets (CaCl_2_, 110.98 g/mol), and diammonium hydrogen phosphate ((NH_4_)_2_HPO_4_, 132.06 g/mol) were purchased from J.T. Baker (Radnor, PA, USA). Alkaline phosphatase (ALP) colorimetric assay kit (GTX85593) was purchased from GeneTex (Hsinchu, Taiwan). 

### 2.1. Samples Preparation

Prior to ECD, the Ti–6Al–4V substrate was etched in 6.2 M H_2_SO_4_ and 4 M HCl at 60 °C for 1 h to remove any oxide and increase surface roughness. The etched samples were washed by immersing them in deionized water at room temperature in an ultrasonic cleaning system at 37 kHz for 30 min. Then, the samples were dried for 24 h to produce natural oxidation to obtain increased bioactivity compared with pure Ti.

Before electrolyte preparation, 0.042 M Ca(NO_3_)_2_ and 0.025 M (NH_4_)_2_HPO_4_ solutions were prepared in deionized water. The etched Ti–6Al–4V substrate was used as a working electrode, a platinum sheet was the counter electrode, Ag/AgCl was the reference electrode, and HAp precursor salts solution served as the electrolyte. The electrolyte was immersed in an oil bath heated at 65 °C. The hybrid coating was prepared at −0.4, −0.8, −1.2, and −1.6 V constant-voltage ECD for 1 h. After ECD, the coated sample was rinsed with deionized water, dried, and stored at ambient temperature.

For the sample of BMP-2 and HAp precursor salts coated by coprecipitation, a treated HAp solution was used as the electrolyte, in which 10 ng/mL BMP-2 was suspended. Untreated Ti–6Al–4V substrate was used as the working electrode attached to the electrochemical reaction vessel. HAp/BMP-2 was coated to the Ti–6Al–4V surface through multicurrent plating with currents and time durations of 0.09 mA and 50 min, 0.22 mA and 50 min, or 0.47 mA and 30 min, respectively [23]. After HAp/BMP-2 coating, the samples were washed with deionized water and lyophilized. To compare the effect of HAp/BMP-2 coprecipitation by ECD, a control sample using the conventional immersion method was also fabricated. The control sample was firstly coated HAp by ECD, and then immersed in a BMP-2 solution of 10 ng/mL for 24 h.

Cyclic voltammetry (CV) and differential pulse voltammetry were performed on an electrochemical workstation CHI-760D potentiostat (CH Instruments Inc., Austin, TX, USA). For X-ray diffraction (XRD) analysis, the HAp powder was scratched from the Ti–6Al–4V substrate and packed to a flat surface on a sample holder. The XRD analysis of the crystallinity and chemical composition structure of the coatings was carried out using a D2 PHASER X-ray diffractometer (Bruker AXS Inc., Madison, WI, USA; X-ray wavelengths: Cu standard ceramic sealed tube, scan mode: 2 theta scan). XRD data were analyzed using X’Pert High Score (Malvern Panalytical Ltd., Malvern, UK). Scanning electron microscopy (SEM; TM-3030, source type: cartridge filament, accelerating voltage 5 kV, Hitachi, Japan) was used to analyze the surface morphology. Surface energy evaluation was assessed by the sessile drop technique to determine the relationship between porosity and the surface tension of the untreated and treated samples by using a contact angle goniometer (GBX, Digidrop, Dublin, Ireland) to determine the surface hydrophilicity. Thicknesses of the HAp films were measured using P10 Profilometer (KLA-Tencor, Milpitas, CA, USA). 

### 2.2. BMP-2 Release

The samples were immersed in 20 mL of simulated body fluid (SBF) solution (pH 7.4) for 7 days to evaluate the release rate of BMP-2. The SBF solution’s ion concentration was approximately equal or identical to that of human blood plasma at 36.5 °C. The SBF containing BMP-2 was extracted at each time point and replaced every day. The amount of released BMP-2 was assessed using a Quantikine enzyme-linked immunosorbent assay (ELISA) immunoassay. The BMP-2 concentration was determined using the standard curve created by following the precision on its corresponding ELISA kit. Accumulative release (%) was equal to the release amount at each juncture over the total amount encapsulated multiplied by 100.

### 2.3. Cell Test

A commercialized cell, human osteosarcoma (MG-63, ATCC CRL-1427) osteoblast-like cell, was used for the cell test in this study. The cell has been used for testing the bioactivity and biocompatibility of various biomaterials [24,25,26]. MG-63 cells were cultured in Dulbecco’s Modified Eagle’s Medium (DMEM) medium containing 10% heat-inactivated Fetal bovine serum (FBS), 50 U/mL penicillin, and 50 mg/mL streptomycin on a culture dish in a humidified atmosphere of 5% CO_2_ and 95% air at 37 °C. The culture media were replaced every 3 days. The HAp/BMP-2 coprecipitation samples were firstly placed on a 24-well plate, and the cells were seeded on the samples at 1 × 10^4^ cells/well in DMEM medium. Then, 1 mL of the culture medium was added after 6 h of incubation and replaced every 2 days.

After coculture of DMEM with the MG-63 cells, cell viability was determined through the MTT assay. After 1, 4, and 7 days of coculture, 50 μL of MTT reagent was added to the cells, followed by incubation for 4 h, and finally, the medium was removed. After removal, 500 μL of DMSO was added to dissolve the formazan crystals. Then, 100 μL of the formazan crystals was added into individual wells of a 96-well plate, and its absorbance was read on an ELISA reader at a wavelength of 570 nm. The values were calculated as percentages of the control groups (cells not cocultured with the etched Ti–6Al–4V specimen).

To test cell proliferation, MG-63 cells were cultured in 24-well plates with 10 ng/mL of BMP-2. The culture medium was changed every 2 days. After 4 and 7 days, 50 μL of MTT reagent was added to the cells, followed by incubation for 4 h. The medium was removed, and 500 μL of DMSO was added to colorize the solution. Next, 100 μL of the formazan crystals was added into individual wells of a 96-well plate, and absorbance was read on an ELISA reader at a wavelength of 570 nm.

ALP activity was used to determine the cell differentiation and the ability to convert p-nitrophenyl phosphate (pNPP) to p-nitrophenyl, and high ALP activity produces a mineralized bone matrix. MG-63 cells were cultured on the HAp/BMP-2 coprecipitation samples with DMEM in 24-well plates, washed with PBS, and lysed with 3 mL of 0.1% Triton X-100. After 4 days, the seeded cells were collected and a 2–3 times of 30 g/cc of the assay buffer was added to the collected cells. Then, cell lysates were collected after being centrifuged at 13,000 rpm for 5 min at 4 °C. A total of 80 μL of cell lysate was transferred to a 96-well plate, and 50 μL of pNPP was added to each well. After 1 h incubation at room temperature, 20 μL was taken from the stock solution and read at 405 nm. The ALP activity was determined as the amount of nitrophenol produced normalized to the total cell DNA content.

## 3. Results and Discussion

**Figure 1:**
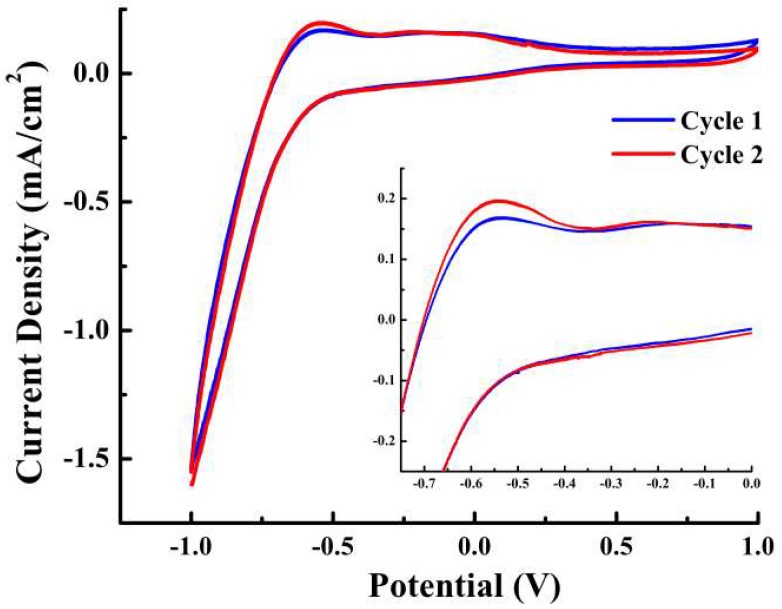
Cyclic voltammetry data of etched Ti substrate with a scan rate of 100 mV/s. Inset is the enlarged curve ranging from −0.75 to 0 V. A significant peak was observed at approximately −0.55 V.

Figure 1 shows the CV data of the etched Ti–6Al–4V substrate obtained in a typical three-electrode electrochemical cell over a range of 1 to −1.0 V, and the inset of Figure 1 (enlarged CV) shows a well-observed significant peak. The electrochemical process was scanned at 100 mV/s at 65 °C in a solution containing 0.042 M Ca(NO_3_)_2_ + 0.025 M (NH_4_)_2_HPO_4_ + 3 M KCl. The electrochemical reaction of electroactive species in the bath solution began at approximately −0.5 V, simplified as a redox reaction between the voltage range of −0.8 to −0.4 V.

The current density tended to increase slowly between −0.4 and −0.8 V, representing the nucleation activity of the HAp on the Ti–6Al–4V surface as well as electron transfer. Electron transfer determined the electrochemical process. The mechanism of HAp during ECD is very complex. The redox reaction can be summarized as calcium ions combining with an acid phosphate group to produce CaHPO_4_·2H_2_O precipitation [27]:

Ca^2+^ + HPO_4_^2−^ + 2H_2_O → CaHPO_4_·2H_2_O + 2H_2_O (1)

Then, CaHPO_4_·2H_2_O is converted from the precursor phase into hydroxyapatite in alkaline solution [28]:

5CaHPO_4_·2H_2_O + 6OH^−^ → Ca_5_(PO_4_)_3_OH + 2PO_4_^3−^ + 15H_2_O (2)

At voltage from −0.8 to −1.0 V, the current density increased rapidly, corresponding to the deposition of phosphoric acid, which was formed through electron transfer and mass transfer. The hydrogen reaction voltage was higher than −2.2 V, as reported by other studies, indicating that it was the dominant reaction [29]. Scans revealed the deposition of HAp on Ti–6Al–4V surface from numerous bubbles at the surface, which can occur during HAp deposition on the etched Ti–6Al–4V samples not subjected to hydrogen precipitation interference. In this study, four voltages (−0.4, −0.8, −1.2, and −1.6 V) were selected from a standard desirable potential range for favorable HAp coating (−0.7 to −2.2 V) [29]. Findings confirmed that a voltage of −0.8 V is preferable for the ECD of HAp on Ti–6Al–4V substrate.

**Figure 2:**
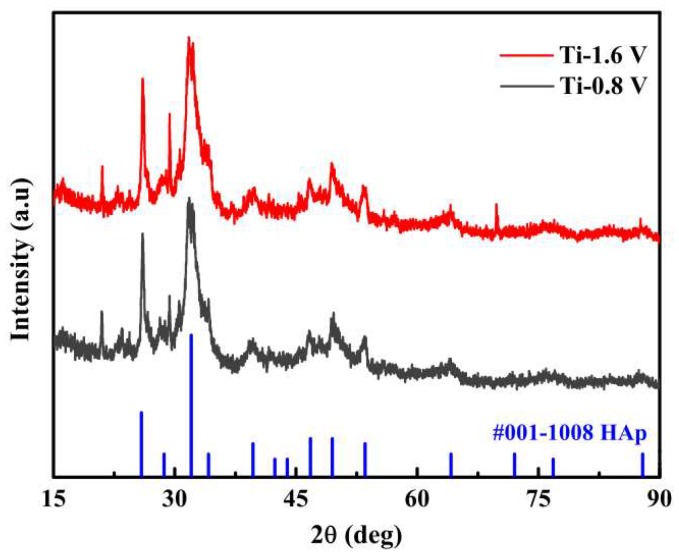
X-ray diffraction patterns of the hydroxyapatite (Hap) coatings by using electrochemical deposition ECD at −1.6 and −0.8 V.

Figure 2 illustrates the XRD patterns of the HAp powder at electrodeposition voltages of −1.6 and −0.8 V. The indexed evaluation revealed that the deposited material at diffraction angles of 26.15°, 28.56°, and 31.88° demonstrated equal or similarly high diffraction peaks with HAp. This confirmed that the HAp remained crystallized following deposition on the Ti–6Al–4V substrates and had the same chemical composition as the HAp powder. The Ti-0.4 V sample did not demonstrate any Hap diffraction peak because the phosphoric acid was insufficient, leading to the deposited HAp being less crystalline.

**Figure 3:**
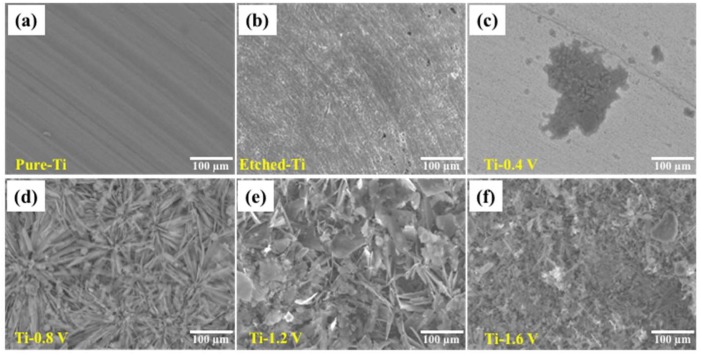
Scanning electron microscopy images of the HAp coatings after ECD on (**a**) pure Ti, (**b**) etched Ti, (**c**) Ti-0.4 V, (**d**) Ti-0.8 V, (**e**) Ti-1.2 V, and (**f**) Ti-1.6 V.

The surface morphologies of the HAp coatings on the Ti–6Al–4V substrate were assessed by SEM and are shown in Figure 3 for pure Ti (Figure 3a), etched Ti (Figure 3b), Ti-0.4 V (Figure 3c), Ti-0.8 V (Figure 3d), Ti-1.2 V (Figure 3e), and Ti-1.6 V (Figure 3f). In Figure 3a, the pure Ti substrate exhibits a fairly smooth surface, whereas the etched Ti and Ti-0.4 V have coarse and tiny pores because of uneven coatings of HAp. Figure 3d–f show significant changes to the coating surface on the Ti–6Al–4V substrate, demonstrating obvious crystallinity. The coatings in Figure 3d show needlelike crystals, those in Figure 3e,f were more compact and uniformly distributed over the coating area, flaky, and covering the entire coating surface. The observed morphology of samples Ti-0.8 V, Ti-1.2 V, and Ti-1.6 V was very similar to the previous report [23], indicating the successful deposition of HAp on the Ti–6Al–4V substrate after applying electrode potentials of −0.8 V, −1.2 V, and −1.6 V. The thickness of the HAp films measured by profilometer were ~0, 20.06 ± 3.02, 35.55 ± 4.71, and 39.89 ± 3.08 μm for Ti-0.4 V, Ti-0.8 V, Ti-1.2 V, and Ti-1.6 V samples, respectively.

**Figure 4:**
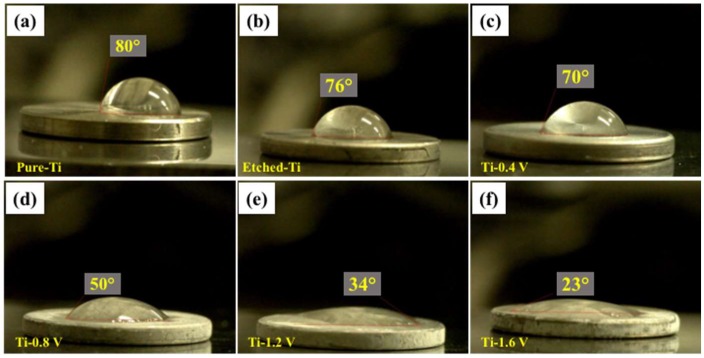
Contact angles on different samples: (**a**) pure Ti, (**b**) etched Ti, and deposited HAp (**c**) Ti-0.4 V, (**d**) Ti-0.8 V, (**e**) Ti-1.2 V, and (**f**) Ti-1.6 V.

Surface hydrophilicity is illustrated in Figure 4 for the untreated (pure Ti), etched, and HAp-deposited on Ti–6Al–4V surface under different ECD potential conditions. The hydrophilicities or water contact angles for each of the samples are given, with the pure Ti (Figure 4a) exhibiting a water contact angle of 80°, and the etched Ti (Figure 4b) exhibiting an angle of 76°, similar to the pure Ti. However, all treated and HAp-deposited on Ti–6Al–4V surface had lower or decreasing contact angles: 70°, 50°, 34°, and 23°. The higher hydrophilicity of the treated Ti–6Al–4V surface could be attributed to the positive and negative charges of the calcium and phosphate ions [30]. The contact angle decreased significantly, demonstrating that a higher surface roughness leads to a lower contact angle, which indicates that osseointegration was more likely to occur. Therefore, Ti–6Al–4V surface with higher hydrophilicities showed a significant enhancement of cell attachment and proliferation, which is attributed to the surface charges obtained from the ion immobilization [31,32].

**Figure 5:**
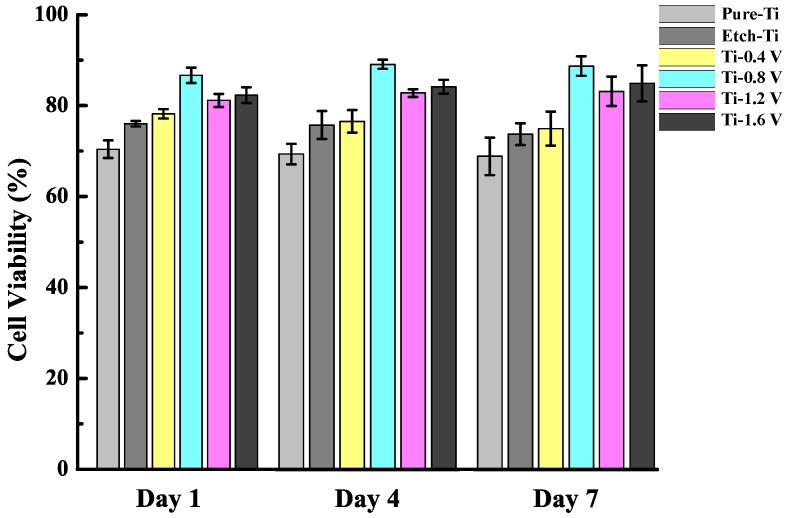
Viabilities of MG-63 cells cultured on treated Ti samples. The cell proliferation rate increased significantly with Ti-0.8 V, Ti-1.2 V, and Ti-1.6 V.

Figure 5 shows the viability of MG-63 cells cocultured with HAp and DMEM for 1, 4, and 7 days. Pure-Ti and etched-Ti were used as the control groups. The etched-Ti sample was confirmed to have increased cell activity compared with the pure-Ti sample, indicating the enhancement of MG-63 cell proliferation. The cell viabilities obtained from the etched-Ti sample after 1, 4, and 7 days revealed decreased cell activity. Moreover, the cell proliferation rate increased significantly with Ti-0.8 V, Ti-1.2 V, and Ti-1.6 V, all being higher than the other three groups. This result indicates that HAp application increased cell viability. The cell viability of the Ti-0.4 V sample is much lower than that of other ECD samples, which is due to insufficient coating shown in Figure 3.

**Figure 6:**
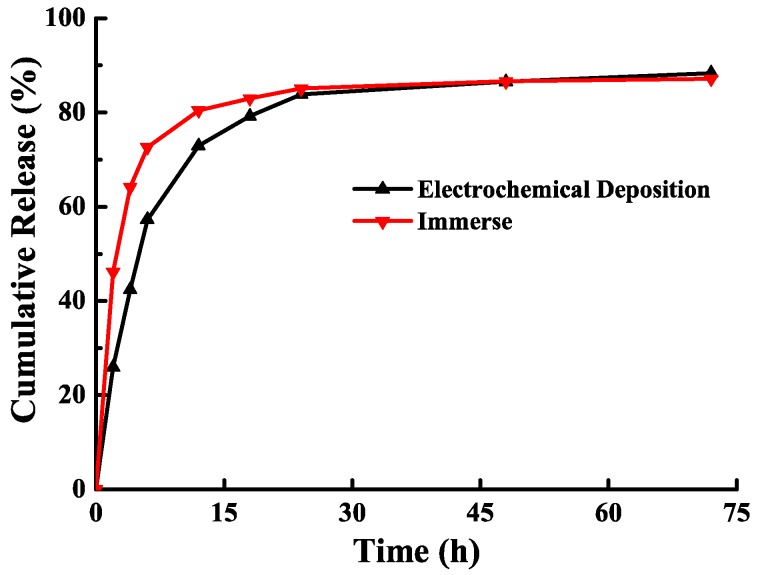
Cumulative release of BMP-2 from the incorporated ECD hybrid coating and immersion samples.

The BMP-2 release rates of the samples using ECD hybrid coating and immersion methods are shown in Figure 6. The BMP-2 release of the ECD sample was slowly released through soaking, and it continued to be released slowly for over 48 h, whereas the sample using immersion method could sustain within 24 h. Table 1 lists the drug loading and release rate of BMP-2 from ECD and immersion samples. The drug-loading rate of the ECD sample was 41.6%—1.5 times higher than immersion method. The release rate was 72.9%, which was lower than that of the sample produced via the immersion method (80.4%). Thus, the control dose can be lowered to improve the release effect, thus providing a safer technique.

**Figure 7:**
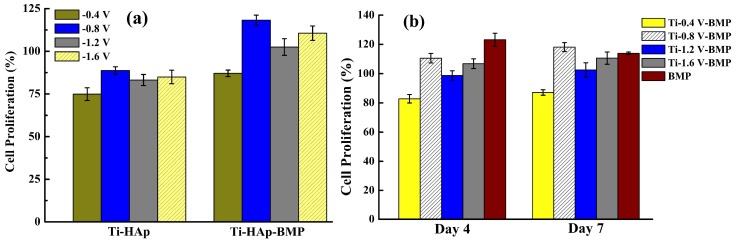
Cell proliferation sequential release of (**a**) Ti–HAp and Ti–HAp–BMP coprecipitation samples after 4 days; (**b**) samples under different ECD potential conditions after 4 and 7 days. The Ti-0.8 V BMP sample showed a better and continuously increasing cell proliferation rate for up to 7 days.

Figure 7a shows the cell proliferation sequential release of Ti–HAp and loaded Ti–HAp–BMP after coculture with cells for 4 days. It is obvious that BMP-2 promoted cell proliferation and was successfully loaded onto the Ti–6Al–4V substrate, and the MG-63 cells were cocultured with the etched samples with different potential settings. The cell proliferation led to the BMP-2 activity being maintained in the process of drug loading, which effectively achieved cell growth. The Ti-0.8 V Ti–HAp–BMP possessed the highest cell proliferation efficiency, which means the Ti-0.8 V Ti–HAp–BMP sample is more favorable for cell growth. Figure 7b shows the cell proliferation of MG-63 cells cocultured with Ti–HAp–BMP for 4 and 7 days, and samples with BMP-2 only are also shown as the control. The BMP (control) sample demonstrated a good cell proliferation effect after 4 days, but the cell proliferation decreased after 7 days. On the other hand, the cell proliferation rate of the Ti-0.8 V BMP sample was continuously increasing after 7 days, demonstrating that drug loading could be sustained and affect cell proliferation. As mentioned, cell proliferation can occur as a result of a high concentration of calcium ions, but the underlying mechanism remains unknown [33]. Nonetheless, the cell proliferation occurring on Ti–HAp–BMP could be a result of the increased amount of Ca on the Ti surface boosting the local calcium ion concentration.

**Figure 8:**
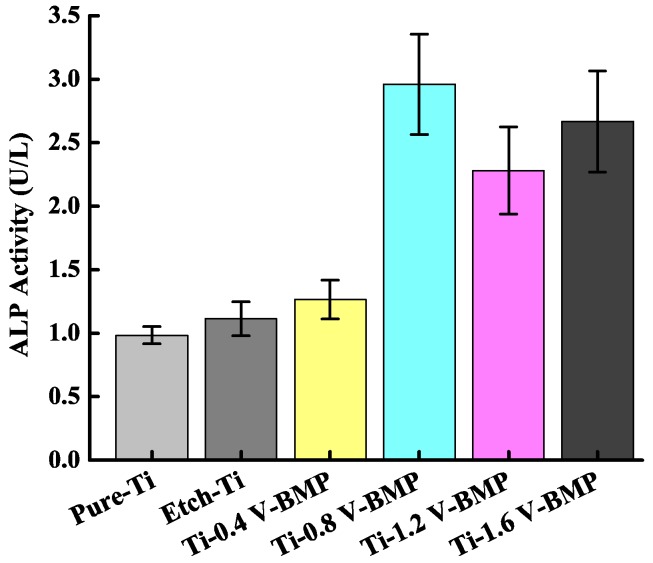
In vitro cell alkaline phosphatase (ALP) activity of MG-63 cells cultured on HAp/BMP-2 coprecipitation samples.

Figure 8 shows the ALP activities of MG-63 cells cultured on Ti–6Al–4V substrate from the incorporated ECD hybrid coating. The Ti-0.8 V BMP sample exhibited the highest cell differentiation (ALP activity). Ti-1.2 V BMP and Ti-1.6 V BMP were insignificantly different from pure-Ti BMP, etched Ti BMP, and Ti-0.4 V BMP. Thus, the higher the ALP activity, the higher the cell differentiation ability, confirming that Ti-0.8 V BMP, Ti-1.2 V BMP, and Ti-1.6 V BMP have more favorable effects on cell differentiation. The interactions between cells are crucial to achieving efficient differentiation between pure Ti and BMP-2. When BMP-2 is cultured on microdomains at various cell densities, the ALP activity increases with increased cell numbers [32].

## 4. Conclusions

BMP-2 and HAp hybrid coatings were successfully deposited on Ti–6Al–4V (Ti–HAp) by ECD. XRD patterns confirmed the coatings of HAp crystals. The surface morphology of the HAp coatings exhibited notable changes following immersion for several days in SBF solution. The Ti–6Al–4V incorporated HAp by ECD hybrid coating at 0.8 V exhibited an increase of the BMP-2 bioactivity, including their contact angle, cell differentiation and proliferation, and ALP activity, which was confirmed through coculture. The results indicated that co-immobilized HAp ions synergistically activated the responses of etched Ti. This simple and cost-effective technique could be a useful and efficient approach using ECD to achieve excellent osteoconductivity and osteoinduction, which could improve osseointegration in biomedical applications.

## Figures and Tables

**Table 1:** Drug loading and release rates of BMP-2 from ECD and immersion samples.

Efficiency	ECD (%)	Immersion (%)
Loading	41.6	26.2
Release (12 h)	72.9	80.4
